# *De Novo* Transcriptome Sequencing of the Orange-Fleshed Sweet Potato and Analysis of Differentially Expressed Genes Related to Carotenoid Biosynthesis

**DOI:** 10.1155/2015/843802

**Published:** 2015-11-15

**Authors:** Ruijie Li, Hong Zhai, Chen Kang, Degao Liu, Shaozhen He, Qingchang Liu

**Affiliations:** Beijing Key Laboratory of Crop Genetic Improvement/Laboratory of Crop Heterosis and Utilization, Ministry of Education, China Agricultural University, Beijing 100193, China

## Abstract

Sweet potato,* Ipomoea batatas* (L.) Lam., is an important food crop worldwide. The orange-fleshed sweet potato is considered to be an important source of beta-carotene. In this study, the transcriptome profiles of an orange-fleshed sweet potato cultivar “Weiduoli” and its mutant “HVB-3” with high carotenoid content were determined by using the high-throughput sequencing technology. A total of 13,767,387 and 9,837,090 high-quality reads were produced from Weiduoli and HVB-3, respectively. These reads were* de novo* assembled into 58,277 transcripts and 35,909 unigenes with an average length of 596 bp and 533 bp, respectively. In all, 874 differentially expressed genes (DEGs) were obtained between Weiduoli and HVB-3, 401 of which were upregulated and 473 were downregulated in HVB-3 compared to Weiduoli. Of the 697 DEGs annotated, 316 DEGs had GO terms and 62 DEGs were mapped onto 50 pathways. The 22 DEGs and 31 transcription factors involved in carotenoid biosynthesis were identified between Weiduoli and HVB-3. In addition, 1,725 SSR markers were detected. This study provides the genomic resources for discovering the genes involved in carotenoid biosynthesis of sweet potato and other plants.

## 1. Introduction

Sweet potato,* Ipomoea batatas* (L.) Lam., is an important food crop widely cultivated in the world, especially in the tropics, subtropics, and some temperate zones of the developing countries [1, 2]. This crop is also used to produce alcohol and various antioxidants such as anthocyanin and carotenoids [3, 4]. The storage roots of orange-fleshed sweet potato are rich in beta-carotene, a precursor of vitamin A [5]. High carotenoid content has become one of the most important objectives in sweet potato breeding [6]. Sweet potato is an autohexaploid (2*n* = 6*x* = 90) and its estimated genome size is 2.4 Gb [7]. The genome data sources for sweet potato are important for gene discovery due to its complex genome.

Carotenoids are widely produced in plants, algae, fungi, and bacteria and provide potent nutritional benefits to humans because their bodies are unable to synthesize carotenoids independently [8, 9]. The necessity of this nutritional component has caused scientists to try to increase the carotenoid content of crops through different approaches. In plants, carotenoids are synthesized through a series of chemical reactions including condensation, dehydrogenation, cyclization, hydroxylation, and epoxidation. To date, a number of genes involved in the carotenoid biosynthesis have been cloned from several plants and their overexpression was found to significantly increase carotenoid levels in canola seeds [10], tomato fruits [11], and rice seeds [12, 13]. Several carotenoid biosynthesis-associated genes have also been isolated from sweet potato [6, 14–17]. However, the molecular mechanisms regulating flux through the pathway are unclear though carotenoid synthesis is well characterized.

Genomic approaches have been used for discovering the important genes involved in plant secondary metabolism pathways. However, the genome of sweet potato is still unavailable. Transcription sequencing is an efficient way for discovering and characterizing novel enzymes and transcription factors from sweet potato. Transcriptome sequencing of sweet potato has provided an important transcriptional data source for studying storage root formation, flower development, and anthocyanin biosynthesis of this crop [7, 18–22]. Here, we performed* de novo* transcriptome sequencing of the orange-fleshed sweet potato by Illumina paired-end (PE) RNA sequencing technology and analyzed differentially expressed genes related to carotenoid biosynthesis.

## 2. Materials and Methods

### 2.1. Plant Materials

The orange-fleshed sweet potato cultivar “Weiduoli” and its high carotenoid mutant “HVB-3” were used in this study. Weiduoli is a commercial cultivar with carotenoid content of 9.02 mg/100 g (FW) and *β*-carotene content of 7.70 mg/100 g. In HVB-3, the contents of carotenoid and *β*-carotene are up to 21.42 mg/100 g and 19.95 mg/100 g, respectively. The storage roots of both materials were harvested after 110 days of planting, cleaned with sterilized water, and cut into 5 mm × 5 mm pieces. The collected samples were immediately frozen in liquid nitrogen and stored at −80°C for RNA extraction.

### 2.2. RNA Extraction

Total RNA from storage roots of Weiduoli and HVB-3 was extracted using the RNAprep Pure Plant Kit (Tiangen Biotech, Beijing, China). To avoid the contamination of genomic DNA, the extracted RNA was treated with DNase I (Takara, Dalian, China) for 4 h at 37°C. The quality of RNA was examined using 1% agarose gel before proceeding. Total RNA was quantified by using a Nanodrop spectrophotometer (Thermo Nanodrop Technologies, Wilmington, DE, USA). Both the A260/280 and A260/230 ratios were checked to ensure the purity of the total RNA and 10 *μ*g RNA was used for Illumina paired-end (PE) sequencing.

### 2.3. cDNA Library Construction and Illumina Sequencing

Using magnetic beads with oligo (dT), poly-A mRNA was enriched from total RNA to construct a cDNA library for RNA sequencing. The enriched mRNA was broken into short fragments by adding fragment buffer. Using these short fragments, the first-strand of cDNA was synthesized by random hexamer primers. Then, using DNA polymerase I and RNase H (Tiangen Biotech, Beijing, China), the second-strand of cDNA was synthesized. After purification with a QiaQuick PCR extraction kit (Qiagen, Valencia, CA, USA), the cDNA fragments were resolved in elution buffer (EB) for end reparation and the addition of a poly(A) tail. Sequencing adapters were connected to the short fragments. These products were purified by agarose gel electrophoresis and suitable fragments (about 180 bp) were isolated as templates for PCR amplification. The cDNA library was constructed for sequencing by 2 × 100 PE using Illumina HiSeq 2000.

### 2.4. Raw Sequence Processing and* De Novo* Assembly

To obtain high quality reads for* de novo* assembly, the raw reads from RNA-seq were cleaned by removing adaptor sequences, empty reads, low quality reads (with ambiguous sequences “*N*”), and reads with more than 10%  *Q* < 20 bases (*Q* = −10 × lgE). The clean reads from the two libraries were assembled together with the Trinity software [23]. The reads were assembled into the contigs with the Inchworm program. The minimally overlapping contigs were clustered into sets of connected components by the Chrysalis program, and then the transcripts were constructed by the Butterfly program. The transcripts were clustered by similarity of correct match length beyond the 80% of longer transcript or 90% of shorter transcript using multiple sequence alignment tool—BLAST [24]. Taking the longest transcript as the unigene of each cluster, these unigenes formed into the nonredundant unigene database.

### 2.5. Analysis of Differentially Expressed Genes (DEGs)

The expression of unigenes in Weiduoli and HVB-3 was calculated according to the RPKM method (reads per kb per million reads) described by Mortazavi et al. [25]. The IDEG6 software [26] was used to identify DEGs in the two libraries. The results of all statistical tests were corrected for multiple testing with the Benjamini-Hochberg false discovery rate (FDR < 0.01) and an absolute value of log2 ratio >1 was used to determine significant differences in gene expression.

### 2.6. Functional Annotation and Classification of DEGs

In order to deduce the correct transcription direction and protein sequences coded by DEGs, a BLASTX search was performed against the National Center for Biotechnology Information (NCBI) nonredundant (Nr) protein database (http://www.ncbi.nlm.nih.gov), the Swiss-Prot protein database, (http://www.expasy.ch/sprot), the Kyoto Encyclopedia of Genes and Genomes (KEGG) pathway database (http://www.genome.jp/kegg), Pfam database, and Cluster of Orthologous Groups (COG) database (http://www.ncbi.nlm.nih.gov/COG) with a typical cut-off *E* value of 10^−5^. Gene ontology (GO) was applied with the Blast2GO program to obtain annotation of DEGs [27]. The WEGO software was then used to perform GO functional classification of DEGs. DEGs were annotated with corresponding Enzyme Commission (EC) numbers using BLASTX alignments against KEGG with a cut-off *E* value of 10^−5^. Gene names were assigned to each DEG based on the best BLAST hit (highest score). Searches were limited to the first 10 significant hits for each query to increase computational speed.

### 2.7. SSR Detection

SSRs were detected among the unigenes with length >1,000 bp using the software MISA (http://pgrc.ipk-gatersleben.de/misa/). A total of 6 types of SSRs were investigated, including mono-, di-, tri-, tetra-, penta-, and hexanucleotide repeats.

## 3. Results

### 3.1. Transcriptome Sequencing and* De Novo* Assembly

**Figure 1:**
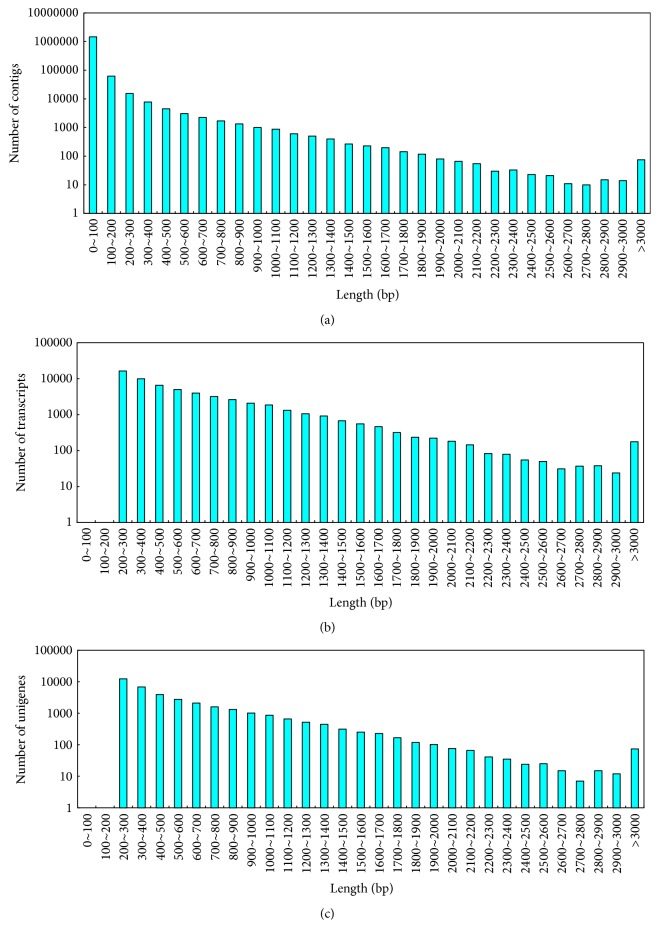
Overview of the sweet potato transcriptome assembly. (a) Size distribution of contigs; (b) size distribution of transcripts; and (c) size distribution of unigenes.

Illumina HiSeq 2000 was used to determine the transcriptome profiles of sweet potato. After removing adaptor sequences and unknown or discarding low quality reads, 13,767,387 and 9,837,090 high-quality reads were obtained from Weiduoli and HVB-3, respectively (Table 1). With the Trinity assembly software, the high-quality reads were assembled into 1,557,001 contigs with an average length of 58 bp and N50 length of 58 bp. These contigs were assembled into 58,277 transcripts with an average length of 596 bp and N50 length of 767 bp. The transcripts were further clustered into 35,909 unigenes with an average length of 533 bp and N50 length of 669 bp (Table 1). The length distributions of contigs, transcripts, and unigenes were shown in Figure 1.

### 3.2. Identification and Functional Annotation of DEGs

**Figure 2:**
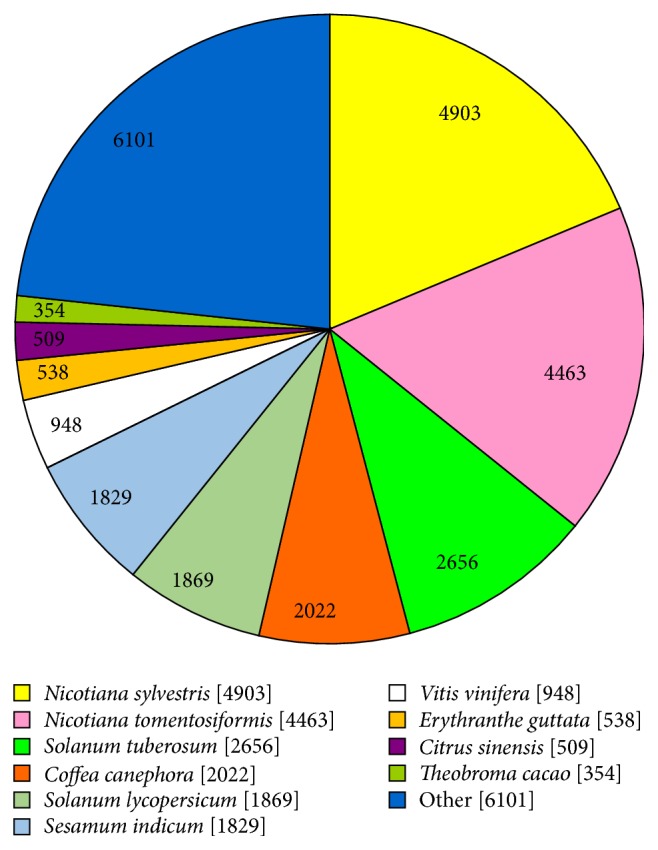
Species distribution of the top BlastX hits for each unigene in the Nr database.

According to the BLASTX results, most of the unigenes had homologous proteins in the Nr protein database. Interestingly, 4,903 (18.71%) and 4,463 (17.03%) unigenes showed significant homology with sequences of* Nicotiana sylvestris* and* Nicotiana tomentosiformis*, respectively (Figure 2). Furthermore, 14,316 (54.21%) unigenes had significant matches in the Pfam database, and 17,058 (64.59%) unigenes had similarity to proteins in the Swiss-Prot database.

**Figure 3:**
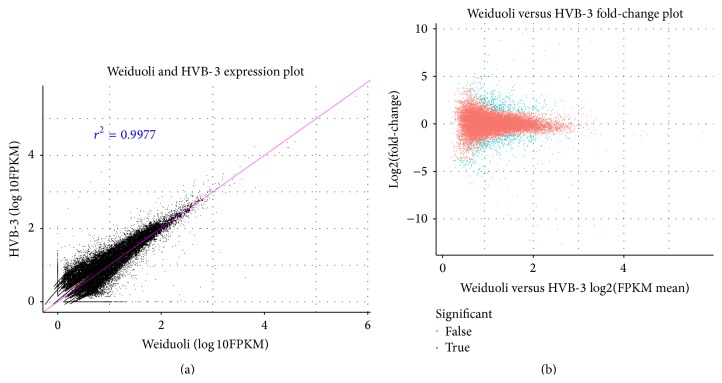
Comparative analysis of gene expression in Weiduoli and HVB-3. (a) A scatter plot of RPKM logarithmic values in libraries of Weiduoli and HVB-3. Each dot represents the RPKM value of a specific gene. The greater deviation from the diagonal slope shows a greater expression level of the gene in the corresponding material. (b) A scatter plot of the ratio of RPKM logarithmic numerical values of genes in Weiduoli and HVB-3. This plot graphically represents genes differentially expressed between Weiduoli and HVB-3. Blue dots represent genes that had significant difference and red dots represent genes where no significant difference was observed between Weiduoli and HVB-3.

The expression of unigenes was calculated according to the RPKM method. A total of 35,909 unigenes had detectable levels of expression in Weiduoli and HVB-3 (Figure 3(a)). Using the IDEG6 software, a total of 874 genes were found to be differentially expressed between HVB-3 and Weiduoli, and 401 of them were upregulated and 473 were downregulated in HVB-3 compared to Weiduoli (Figure 3(b)). A total of 697 DEGs were annotated against the public databases and 94.12% of them were mapped to the Nr library, suggesting that most of the DEGs can be translated into proteins. The mapping rate of DEGs against the Swiss-Prot protein database was 67.58%. The overall functional annotation is listed in Table 2.

**Figure 4:**
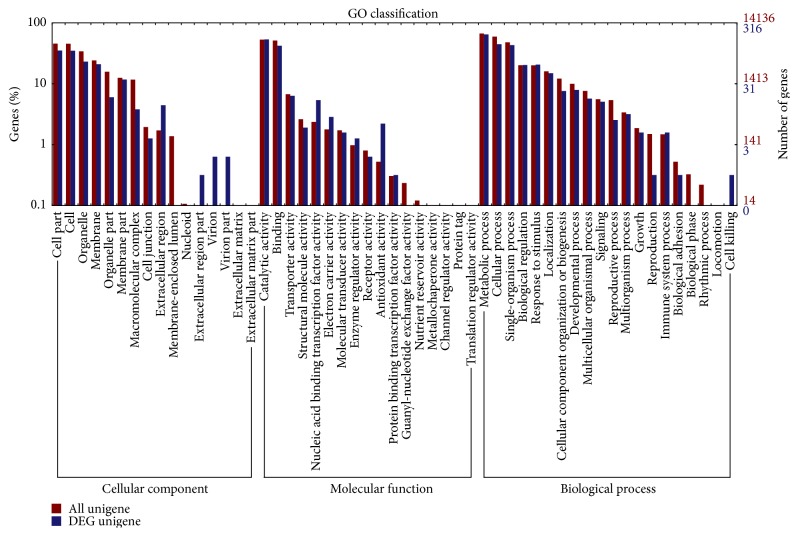
GO classification of unigenes in transcriptomes of Weiduoli and HVB-3. The red bars represent all the unigenes and the blue bars represent the DEGs.

**Figure 5:**
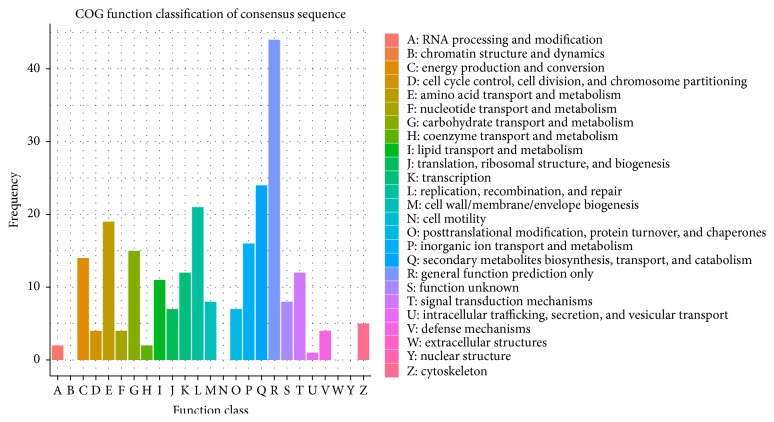
COG-based functional classification of DEGs between Weiduoli and HVB-3.

A total of 14,136 unigenes were classified into three categories, cellular component, biological progress, and molecular function, through GO analysis (Figure 4). In all, 316 DEGs were classified into three categories, 149 with cellular component, 235 with biological progress, and 245 with molecular function, through GO analysis (Figure 4). The GO analysis revealed that most of the DEGs were involved in catalytic activity and metabolic process. Compared with the COG database, 240 DEGs were subdivided into 22 COG classifications, including secondary metabolite biosynthesis, transport, and catabolism, signal transduction mechanisms, replication, recombination, and repair, amino acid transport and metabolism, inorganic ion transport and metabolism, carbohydrate transport and metabolism, energy production and conversion, transcription, and lipid transport and metabolism (Figure 5).

**Figure 6:**
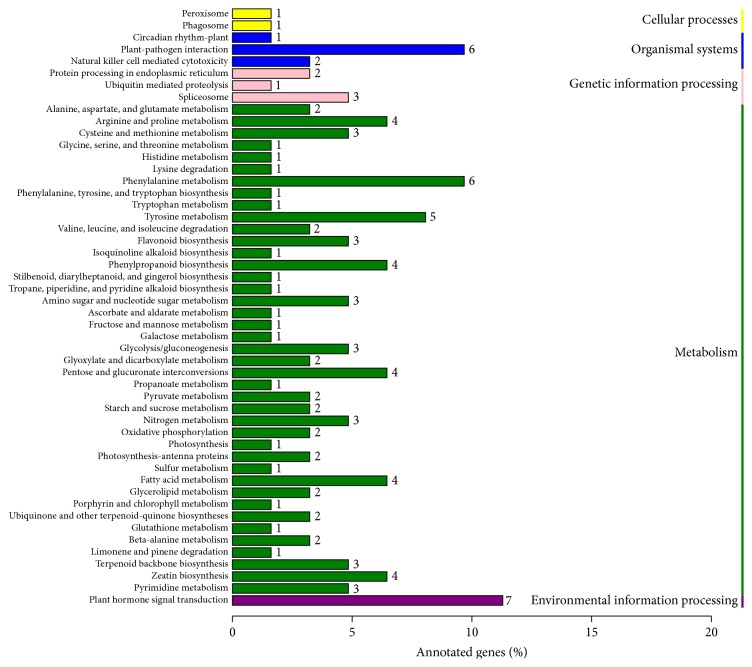
KEGG-based functional classification of DEGs between Weiduoli and HVB-3. Numbers beside each bar represent the actual number of DEGs classified in that descriptive term.

Carotenoid biosynthesis which belongs to the secondary metabolisms is a dynamic and complex process catalyzed by a series of enzymes. Functional category analysis revealed that the DEGs were involved in a number of important pathways, including metabolite biosynthesis and signal transduction mechanisms (Figure 6), similar to the results of GO and COG analyses. According to the KEGG pathway enrichment results, 62 DEGs were assigned to the 50 pathways. The most noticeable pathways were terpenoid backbone biosynthesis and fatty acid metabolism. As shown in Table 3, 22 DEGs were found to be directly or indirectly involved in carotenoid biosynthesis. These 22 DEGs encoded geranylgeranyl pyrophosphate synthase (GGPS), geranylgeranyl diphosphate reductase (GGPR), dehydrodolichyl diphosphate synthase (DHDDS), alcohol dehydrogenases homologous, aldehyde dehydrogenase, alcohol dehydrogenase, long chain acyl-CoA synthetase, and 15 cytochrome P450, respectively (Table 3). Interestingly, several important transcription factors, including NAC, MYB, AP2/ERF, Zifc fingers, WRKY, bZIP, and ARF, were found to be significantly upregulated in HVB-3 compared to Weiduoli (Table 3).

### 3.3. SSR Markers

The MISA was used to search for SSRs. A total of 1,725 potential cDNA-derived SSRs (cSSRs) were identified from 4,061 unigenes. Most of them were mononucleotide repeats (1,005), followed by trinucleotide repeats (388), dinucleotide repeats (298), and tetranucleotide repeats (26), with only a small portion of pentanucleotide (4) and hexanucleotide repeats (4). There were 323 sequences containing more than 1 cSSR and 125 cSSRs present in compound formation.

## 4. Discussion

In nonmodel plants, it is difficult to identify the candidate genes involved in complex biosynthetic pathways due to the limited availability of genomic data [28, 29]. With high-throughput transcriptome sequencing technology, this limitation has been overcome, as it can generate large amounts of data on genome wide transcription [30]. Several sweet potato transcriptomes have been sequenced, which provide an important data source for storage root formation, flower development, and anthocyanin biosynthesis of this crop [7, 18–22].

Carotenoids are widely distributed pigments in plants and play an important role as light-harvesting pigments in most photosynthetic organisms [31, 32]. In many photosynthetic and nonphotosynthetic organisms, the carotenoid biosynthesis pathway has been well studied and a series of genes involved in this pathway have been cloned and characterized. However, the molecular mechanisms regulating carotenoid synthesis have not been well understood.

The storage roots of orange-fleshed sweet potato typically have a high carotenoid content [5]. In the present study, the transcriptomes of orange-fleshed sweet potato cultivar “Weiduoli” and its high carotenoid mutant “HVB-3” were sequenced on the Illumina HiSeq 2000 sequencing platform, and 13,767,387 and 9,837,090 high-quality reads were produced from Weiduoli and HVB-3, respectively. A total of 35,909 unigenes were harvested from Weiduoli and HVB-3 (Table 1). There were 874 DEGs between HVB-3 and Weiduoli, 401 of which were upregulated and 473 were downregulated in HVB-3 compared to Weiduoli (Figure 3(b)).

The present results showed that the 22 DEGs related to carotenoid biosynthesis existed between Weiduoli and HVB-3 (Table 3). GGPS, GGPR, and DHDDS are involved in terpenoid backbone biosynthesis. GGPS is the key enzyme of carotenoid biosynthesis. Transgenic kiwifruit plants expressing GGPS exhibited the increased *β*-carotene content [33]. GGPR converts geranylgeranyl diphosphate (GGPP), the precursor for carotenoid biosynthesis, to phytyl diphosphate in the tocopherol and chlorophyll biosynthetic pathways. DHDDS is involved in the biosynthesis of isoprenoids, which are the precursors of carotenoid biosynthesis. Four genes encoding alcohol dehydrogenases homologous, aldehyde dehydrogenase, alcohol dehydrogenase, and long chain acyl-CoA synthetase, respectively, are involved in fatty acid metabolism. The biosynthesis of carotenoids and fatty acids requires a common precursor from pyruvate [34]. The 15 DEGs were found to encode the cytochrome P450 family (Table 3).* P450CYP707A* encoding ABA 8′-hydroxylases and* LUT1* encoding cytochrome P450-type monooxygenase (CYP97C1) have been proved to regulate carotenoid biosynthesis in* Arabidopsis* [35, 36]. Thus, it is thought that these DEGs may play important roles in carotenoid biosynthesis of sweet potato.

In the present study, several important transcription factors, including NAC, MYB, AP2/ERF, Zifc fingers, WRKY, bZIP, and ARF, were significantly upregulated in HVB-3 compared to Weiduoli (Table 3). These transcription factors may regulate carotenoid biosynthesis of sweet potato. In plants, transcription factors of different families have been shown to regulate secondary metabolism pathways [37]. NAC proteins are one of the largest families of plant-specific transcription factors [38]. In* Solanum lycopersicum*, a NAC transcription factor (SlNAC4) was shown to function as a positive regulator of carotenoid accumulation [39]. MYB transcription factors were found to participate in a wide range of biological processes [40, 41]. Overexpression of the* Vitis vinifera* MYB5b in tomato resulted in an increased content of *β*-carotene [42].

To date, the genome of sweet potato is still unavailable; therefore much of the research on sweet potato breeding and genetic linkage maps is based on molecular markers [43]. SSRs are widely distributed in both noncoding and transcribed sequences. As transferable markers, SSRs are a useful source for genome analysis due to their abundance, functionality, high polymorphism, and excellent reproducibility [44]. In the present study, a total of 1,725 potential cSSRs, including mono-, di-, tri-, tetra-, penta-, and hexa-SSR, were identified from 4,061 unigenes.

## 5. Conclusion

A total of 35,909 unigenes were identified from the orange-fleshed sweet potato using the high-throughput sequencing technology and most of them are protein-coding genes. There were 874 DEGs between Weiduoli and HVB-3. The 22 DEGs were found to directly or indirectly participate in carotenoid biosyntheses. The 31 important transcription factors were significantly upregulated in HVB-3 compared to Weiduoli. These DEGs and transcription factors may be involved in carotenoid biosynthesis of sweet potato.

## Figures and Tables

**Table 1:** Length distribution of assembled contigs, transcripts, and unigenes from Weiduoli and HVB-3.

Length range	Contig	Transcript	Unigene
0–300	1,531,538 (98.36%)	16,317 (28.00%)	12,325 (34.32%)
300–500	12,342 (0.79%)	16,504 (28.32%)	10,710 (29.83%)
500–1000	9,349 (0.60%)	16,921 (29.04%)	8,813 (24.54%)
1000–2000	3,419 (0.22%)	7,635 (13.10%)	3,671 (10.22%)
2000+	353 (0.02%)	900 (1.54%)	390 (1.09%)
Total number	1,557,001	58,277	35,909
Total length	91,371,759	34,741,399	19,150,802
Mean length	58	596	533
N50 length	58	767	669

**Table 2:** Functional annotation of DEGs between Weiduoli and HVB-3.

Annotation database	Annotation number
COG annotation	167
GO annotation	316
KEGG annotation	83
KOG annotation	290
Pfam annotation	477
Swiss-Prot annotation	471
Nr annotation	656
Total	697

**Table 3:** Differentially expressed genes and transcription factors related to carotenoid biosynthesis between Weiduoli and HVB-3.

Gene ID	Log2 fold-change	FDR	Blast annotation
*Genes*			
c27179.graph_c0	2.28	0	Geranylgeranyl pyrophosphate synthase
c35889.graph_c0	−2.19	6.71*E* − 08	Geranylgeranyl diphosphate reductase
c41187.graph_c4	2.29	3.36*E* − 11	Dehydrodolichyl diphosphate synthase
c21698.graph_c0	−3.96	1.68*E* − 05	Alcohol dehydrogenases homologous
c30364.graph_c1	2.08	0	Aldehyde dehydrogenase
c34785.graph_c0	−2.46	9.95*E* − 05	Alcohol dehydrogenase
c37165.graph_c0	−2.81	3.18*E* − 05	Long chain acyl-CoA synthetase
c26028.graph_c0	−1.58	7.58*E* − 05	Cytochrome P450 82A2
c29728.graph_c0	2.96	0.01	Cytochrome P450 82A4
c29775.graph_c1	−2.20	0	Cytochrome P450 86B1-like
c33165.graph_c0	−2.03	0	Cytochrome p450 CYP82D47-like
c34761.graph_c0	1.77	0	Cytochrome P450 89A2
c34982.graph_c1	−2.66	2.32*E* − 07	Cytochrome P450 82C4
c36936.graph_c0	−2.50	1.63*E* − 06	Cytochrome p450 86B1-like
c38370.graph_c0	2.11	0	Cytochrome P450 CYP72A219
c38487.graph_c0	−1.67	0.01	Cytochrome P450 82C4
c40387.graph_c0	2.38	7.46*E* − 08	Cytochrome P450 83B1
c40784.graph_c0	−1.51	0	Cytochrome P450 CYP736A12
c41229.graph_c0	−2.70	7.30*E* − 13	Cytochrome P450 78A5
c41281.graph_c1	1.22	0.01	Cytochrome P450 76A2
c41491.graph_c0	1.32	0.01	Cytochrome P450 CYP72A219-like
c42321.graph_c0	3.40	0	Cytochrome P450 71A1

*Transcription factors*			
c39444.graph_c0	2.10	5.87*E* − 11	NAC domain-containing protein
c39928.graph_c0	2.14	1.04*E* − 10	NAC domain-containing protein
c41510.graph_c0	1.55	0	NAC domain-containing protein
c4748.graph_c0	3.66	6.33*E* − 06	MYB-like transcription factor
c27997.graph_c0	2.18	0	Ethylene-responsive transcription factor
c32755.graph_c0	3.98	3.64*E* − 11	Ethylene-responsive transcription factor
c32755.graph_c1	3.20	5.42*E* − 09	Ethylene-responsive transcription factor
c34264.graph_c0	1.92	0	Ethylene-responsive transcription factor
c34616.graph_c0	1.27	0.01	Ethylene-responsive transcription factor
c35745.graph_c1	2.64	1.63*E* − 06	Ethylene-responsive transcription factor
c36135.graph_c0	1.32	0	Ethylene-responsive transcription factor
c37886.graph_c1	1.41	0	Ethylene-responsive transcription factor
c39393.graph_c0	1.42	0	Ethylene-responsive transcription factor
c40060.graph_c0	1.45	0	Ethylene-responsive transcription factor
c40767.graph_c1	1.48	8.56*E* − 05	Ethylene-responsive transcription factor
c41013.graph_c0	1.59	4.20*E* − 05	Ethylene-responsive transcription factor
c29615.graph_c0	1.91	0	Zinc finger protein
c30636.graph_c0	1.56	0.01	Zinc finger protein
c33159.graph_c0	1.63	0.01	Zinc finger protein
c33843.graph_c0	1.91	2.63*E* − 05	Zinc finger protein
c34456.graph_c0	1.43	0	Zinc finger protein
c35956.graph_c0	3.37	0	Zinc finger protein
c36278.graph_c0	1.83	0	Zinc finger protein
c36428.graph_c1	1.78	9.34*E* − 06	Zinc finger protein
c33226.graph_c1	2.30	9.80*E* − 05	WRKY transcription factor
c34181.graph_c0	1.59	0.01	WRKY transcription factor
c39751.graph_c0	1.36	0.01	WRKY transcription factor
c35456.graph_c0	1.71	0	bZIP transcription factor
c39175.graph_c0	1.55	0	bZIP transcription factor
c41663.graph_c1	1.95	1.82*E* − 08	bZIP transcription factor
c19855.graph_c0	2.66	0	Auxin response factor
